# Impact of Outpatient Antihypertensive Medication Use on Epinephrine Resistance in Anaphylaxis

**DOI:** 10.7759/cureus.35119

**Published:** 2023-02-17

**Authors:** Christopher S Snider, Shannon L Hasara, Kayla M Wilson, Jesse A Glueck, Andrew R Barbera

**Affiliations:** 1 Department of Pharmacy, Lakeland Regional Health, Lakeland, USA; 2 Department of Emergency Medicine, Lakeland Regional Health, Lakeland, USA

**Keywords:** allergy, emergency department, antihypertensive medication, epinephrine, anaphylaxis

## Abstract

Background: There has been an increase in allergy-related emergency department (ED) visits over the past several years. Underlying cardiovascular disease or respiratory disease and concurrent beta blocker or angiotensin-converting enzyme inhibitor use have been identified as potential risk factors for severe or refractory anaphylactic reactions. Conflicting evidence exists regarding the association between antihypertensive (AH) use and the incidence of refractory anaphylaxis.

Objective: The purpose of this study was to determine the incidence of refractory anaphylaxis in patients presenting to the ED while prescribed select AH medications outpatient.

Methods: This was a retrospective cohort study of all adult and pediatric patients presenting to the ED between February 16, 2021, and August 31, 2021, with a diagnosis of anaphylaxis. The primary objective was to compare the proportion of patients experiencing refractory anaphylaxis that were prescribed versus not prescribed AH medications in the outpatient setting.

Results: A total of 101 patients were treated for anaphylaxis in the ED during the study timeframe with 13 patients in the AH group and 88 patients in the no AH group. There was no difference in the incidence of refractory anaphylaxis between groups (0% vs 9%; p=0.48). Significantly fewer patients in the AH group required any epinephrine doses compared to the no AH group (38% vs 88%; p<0.001).

Conclusions: Outpatient use of select AH medications was not associated with an increased incidence of refractory anaphylaxis in patients presenting to the ED.

## Introduction

Between 2007 and 2015, approximately 10 million emergency department (ED) visits were associated with anaphylaxis and other allergy-related conditions in the United States alone [1]. Anaphylaxis is characterized as a life-threatening hypersensitivity reaction to foods, insect venom, and medications. Historically, antibiotics and analgesics are commonly implicated medication triggers. The 2020 World Allergy Organization guidelines recommend intramuscular epinephrine as first-line therapy while intravenous (IV) histamine-1 and histamine-2 receptor antagonists, IV glucocorticoids, short-acting beta-2 agonists, and IV glucagon are reserved as adjunctive therapies [2].

Extremes of age, underlying cardiovascular or respiratory disease, and concurrent beta blocker (BB), angiotensin-converting enzyme inhibitor (ACE-I), or non-steroidal anti-inflammatory drug use have been identified as potential risk factors for severe or refractory anaphylactic reactions; however, there is conflicting evidence regarding the influence of BB and ACE-I use on the severity of anaphylaxis and the need for repeat epinephrine administration in this setting [2]. In an analysis of The European Anaphylaxis Registry, outpatient BB use led to a numerically higher risk of refractory anaphylaxis episodes (defined as failure to achieve initial control after two or more doses of epinephrine) when compared to non-refractory anaphylaxis episodes [3]. In a retrospective cohort study by Lee and colleagues, BB and ACE-I use were shown to be predictors of increased organ system involvement and hospital admission [4]. A systematic review by Tejedor-Alonso and colleagues found the use of BB and ACE-I therapies increased the severity of reactions, while a retrospective cohort study by White et al. demonstrated that BB use may not have a clinically significant impact on epinephrine dosing among ED patients with anaphylaxis [5,6].

The purpose of this study was to determine the incidence of refractory anaphylaxis in patients presenting to the ED who were prescribed select antihypertensive (AH) medications versus those not prescribed select AH medications outpatient. 

## Materials and methods

This was a single-center, institutional review board-approved, retrospective cohort evaluation of adult and pediatric patients who were treated for anaphylaxis in the ED. Lakeland Regional Health Institutional Review Board issued approval number 1820269-4. The study was conducted at an 864-bed community, tertiary referral center which houses a 165-bed adult ED and 33-bed pediatric ED and sees more than 200,000 patient visits annually.

**Figure 1:**
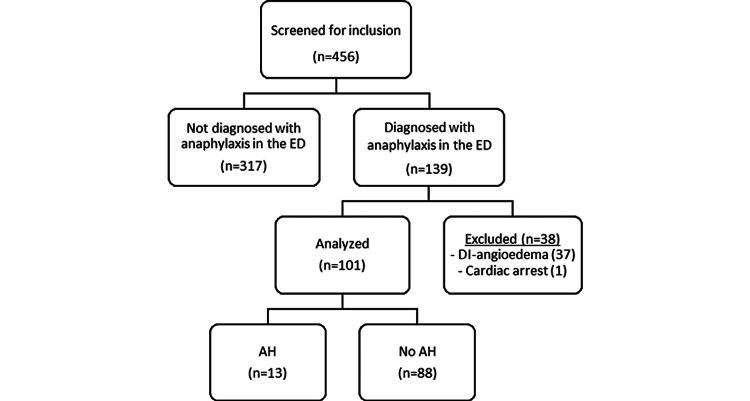
Patient Selection AH: antihypertensive; DI: drug-induced; ED: emergency department

All patients with medications ordered under allergic reaction order sets with a diagnosis of anaphylaxis per ED physician documentation from February 16, 2021, to August 31, 2021, were screened in reverse chronological order for inclusion (Figure 1). Patients were excluded if they presented to the ED in cardiac arrest or with suspected or confirmed drug-induced angioedema. Patients prescribed select AHs (defined as the “AH group”) were compared to patients not prescribed select AHs (defined as the “No AH group”). Patients were included in the AH group if they had any of the medications listed in Table 1 documented in their outpatient fill history or in the ED physician note. A single, designated member of the research team screened all identified patients for inclusion and then extracted data from the electronic health record. Data extracted included age, gender, history of respiratory or cardiovascular disease, outpatient medication fill history, doses of epinephrine administered, route(s) of epinephrine administration, adjunctive medication administration, need for mechanical ventilation, incidence of biphasic reactions, and ED disposition. A biphasic reaction was defined as the administration of epinephrine greater than one hour from initial control for the treatment of recurrent anaphylactic symptoms.

**Table 1:** Definitions

Term	Definition
Alpha antagonist	Alfuzosin, doxazosin, prazosin, silodosin, tamsulosin, terazosin
Angiotensin converting enzyme inhibitor	Benazepril, captopril, enalapril, fosinopril, lisinopril, quinapril, ramipril
Angiotensin receptor blocker	Candesartan, irbesartan, losartan, olmesartan, telmisartan, valsartan
Biphasic reaction	Use of epinephrine greater than one hour from initial control for treatment of recurrent anaphylactic reaction
Cardioselective beta blocker	Acebutalol, atenolol, bisoprolol, metoprolol, nebivolol
Cardiovascular disease	Diagnosis of: coronary artery disease, heart failure, hypertension, and/or stroke
Glucocorticoid	Dexamethasone, hydrocortisone, methylprednisolone, prednisolone, prednisone
Histamine-1 receptor antagonist	Diphenhydramine
Histamine-2 receptor antagonist	Famotidine
Non-cardioselective beta blocker	Carvedilol, labetalol, nadolol, pindolol, propranolol, sotalol
Other bronchodilator	Racemic epinephrine, terbutaline
Outpatient medication use	Medication fill within previous 6 months per prescription insurance claim report or per provider documentation
Refractory anaphylaxis	Requiring treatment with more than one dose of epinephrine to achieve initial control
Respiratory disease	Diagnosis of: asthma, chronic obstructive pulmonary disease, chronic bronchitis, emphysema, cystic fibrosis/bronchiectasis, and/or lung cancer
Select antihypertensive medication	Aliskiren, alpha antagonist, angiotensin converting enzyme inhibitor, angiotensin receptor blocker, cardioselective beta-blocker, non-cardioselective beta-blocker, sacubitril-valsartan
Short-acting beta agonist	Albuterol, levalbuterol, salbutamol

The primary outcome was to compare the proportion of patients experiencing refractory anaphylaxis while prescribed select AH medications versus those not prescribed select AH medications. The secondary outcome was to compare the proportion of patients requiring any epinephrine while prescribed select AH medications versus those not prescribed select AH medications.

Descriptive statistics were used to report baseline characteristics of the study population. A chi-square or Fisher’s Exact Test was utilized to evaluate categorical data. A p-value of <0.05 was deemed statistically significant for all analyses. For quality assurance, an inter-rater blinded to the study objectives was assigned a randomly selected 10% study sample to evaluate repeat epinephrine doses. An inter-rater reliability goal of greater than or equal to 90% agreement was considered acceptable. 

## Results

A total of 456 patients were screened during the study timeframe with 101 patients included in the final analysis. There were 13 patients in the AH group and 88 patients in the no AH group (Figure 1). Baseline demographics are summarized in Table 2. The average age in the no AH group was significantly lower than the AH group (24 years vs. 51 years; p<0.001). Cardioselective BB, non-selective BB, and ACE-I were present in five (38%), three (23%), and two (15%) patients in the AH group, respectively.

**Table 2:** Baseline Demographics *Data expressed as n (%) unless otherwise specified ACE-I: angiotensin converting enzyme inhibitor; AH: antihypertensives; ARB: angiotensin receptor blocker; BB: beta blocker; CI: confidence interval; SD: standard deviation.

	AH (n=13)	No AH (n=88)	95% CI	p-value
					Age (years), mean (SD)	51 (13)	24 (18)	16.7 – 37.3	<0.001
0 – 18	0 (0)	45 (51)	–	–
19 – 65	12 (92)	40 (45)	–	–
66 – 90	1 (8)	3 (3)	–	–
Gender (male)	3 (23)	30 (34)	-17.6 – 29.1	0.43
Past medical history				
Cardiovascular disease	13 (100)	2 (2)	74.5 – 99.5	<0.001
Respiratory disease	4 (31)	18 (20)	-9.5 – 38.7	0.37
Outpatient antihypertensive therapy				
Cardioselective BB	5 (38)	–	–	–
Non-selective BB	3 (23)	–	–	–
Alpha antagonist	2 (15)	–	–	–
ACE-I	2 (15)	–	–	–
ARB	3 (23)	–	–	–

There was no difference in the incidence of refractory anaphylaxis between the AH and no AH groups (0% vs 9%; 95% CI -34.7 - 17.5; p=0.48) (Table 3). Patients in the AH group required significantly fewer doses of epinephrine compared to the no AH group (38% vs 88%; 95% CI 22.4 - 71.3; p<0.001). Administration of adjunctive therapies was similar between groups. No difference was found in discharge disposition from the ED to home (85% vs 89%; 95% CI -9.6 - 31.3; p=0.68). The inter-rater sample met the predefined goal. 

**Table 3:** Clinical Outcomes Data expressed as n (%) unless otherwise specified AH: antihypertensives; CI: confidence interval; H1: histamine-1 receptor; H2: histamine-2 receptor; SABA: short-acting beta agonist; SAMA: short-acting muscarinic agonist; SD: standard deviation

	AH (n=13)	No AH (n=88)	95% CI	p-value
					Epinephrine administration				
Any	5 (38)	77 (88)	22.4 – 71.3	<0.001
Refractory	0 (0)	7 (9)	-34.7 – 17.5	0.48
Epinephrine doses administered				
Zero	8 (62)	11 (12)	22.6 – 71.3	<0.001
One	5 (38)	70 (80)	14.2 – 63.8	0.001
Two	0 (0)	7 (8)	-15.2 – 15.6	0.3
Initial epinephrine dose (mg), mean (SD)	0.3 (0)	0.28 (0.1)	-0.03 – 0.07	0.43
Epinephrine administration route				
Intramuscular	5 (100)	72 (94)	-37.6 – 13.7	0.58
Intravenous	0 (0)	1 (1)	-42.5 – 6.5	0.82
Subcutaneous	0 (0)	4 (5)	-38.6 – 12.3	0.61
Medication administration				
Glucocorticoid	12 (92)	81 (92)	-10 – 26	1
H1 antagonist	12 (92)	77 (88)	-22.2 – 14.7	0.67
H2 antagonist	11 (85)	70 (80)	-22.7 – 19.4	0.67
Intravenous fluid bolus	7 (54)	52 (59)	-20.2 – 31.6	0.73
SABA	4 (31)	18 (20)	-9.5 – 38.7	0.37
Pre-hospital epinephrine	4 (31)	36 (41)	-18.5 – 31	0.49
SAMA	2 (15)	5(6)	-3.92 – 36	0.24
Other bronchodilator	1 (7)	2 (2)	-3 – 30.5	0.3
Epinephrine infusion	0 (0)	0 (0)	–	–
Glucagon	0 (0)	0 (0)	–	–
Mechanical ventilation required	1 (7)	2 (2)	-3 – 30.5	0.30
Experienced biphasic reaction	0 (0)	2 (2)	-20.9 – 7.5	0.61
Emergency department disposition				
Home	11 (85)	78 (89)	-9.6 – 31.3	0.68
Floor	1 (7)	7 (8)	-24.8 – 10.6	0.9
Intensive care unit	1 (7)	3 (3)	-4.3 – 29.5	0.47
Deceased	0 (0)	0 (0)	–	–

## Discussion

Several inflammatory mediators such as bradykinin, histamine, and leukotrienes, are released from mast cells following allergen exposure, resulting in downstream vasodilation, bronchospasm, increased vascular permeability, and decreased peripheral vascular resistance. Consequently, the renin-angiotensin system is activated and endogenous catecholamines are released to counteract any resultant hypotension. When inhibited, ACE (a component responsible for bradykinin breakdown) can trigger the accumulation of bradykinin leading to angioedema, hypotension, and bronchospasm. As such, a patient prescribed chronic AH agents, such as BBs or ACE-Is, may experience a reduced compensatory response to these endogenous catecholamines as well as to exogenous epinephrine leading to intensified or refractory anaphylaxis [4,7].

Francuzik et al. analyzed data from the European Anaphylaxis Registry capturing 42 cases of refractory anaphylaxis (defined as failure to achieve initial control after two or more doses of epinephrine) and 4,820 cases of non-refractory anaphylaxis. Patients reported receiving BBs in the outpatient setting in six cases (14.3%) of refractory anaphylaxis. Although non-significant, BB use increased the risk of experiencing a refractory anaphylactic episode compared to a severe reaction (15.8% vs. 10.4%; p=0.28). Patients with an underlying cardiovascular condition had a non-significant higher risk of experiencing refractory anaphylaxis compared to a severe anaphylactic episode (31.7% vs. 22.8%; p=0.19) [3]. These findings suggest that concomitant BB use and pre-existing cardiovascular disease, respectively, may lead to an increased risk of refractory anaphylaxis. 

A retrospective cohort study of 302 patients with anaphylaxis by Lee and colleagues investigated AH use and the association with anaphylaxis severity. Markers for severity included syncope, hypoxia, hypotension, hospitalization, and three or more organ system involvement. After adjusting for demographic factors, comorbidities, and suspected triggers, AH use was a predictor of hospital admission (OR 4.0; 95% CI 1.9 - 8.4; p=0.0001) and multi-system organ involvement (OR 2.8; 95% CI 1.5 - 5.2; p=0.0008) [4]. Despite these findings, the current study found a non-significant difference in ED disposition.

A meta-analysis by Tejedor-Alonso et al. examining 22,313 anaphylaxis episodes found low to moderate-quality evidence that the use of AH medications increases the severity of anaphylaxis. Despite adjusting outcomes for confounding factors that may increase anaphylaxis severity (e.g., cardiovascular disease, elderly age, BB or ACE-I use), it is still unknown whether these factors, individually or concurrently, lead to more severe anaphylactic reactions [5]. The current study also gathered data regarding age, cardiovascular disease, and AH use, but was unable to find a significant difference in the incidence of refractory anaphylaxis.

White and colleagues conducted a retrospective cohort study analyzing 789 ED patients to describe the association between BBs and the requirement for more than one dose of epinephrine for anaphylaxis. After adjusting for multiple variables, the association of BB use with repeat epinephrine administration was statistically nonsignificant (OR 1.51; 95% CI 0.66 - 3.42; p=.33). Furthermore, there was no significant difference in patients requiring any epinephrine while on a BB versus patients who received no epinephrine (OR 0.73; 95% CI 0.46 - 1.14; p=.17) [6]. The current study aimed to expand upon this association by including a wider variety of AH medications yet revealed similar results.

In this retrospective cohort study of ED patients diagnosed with anaphylaxis, the use of outpatient AH medications did not increase the number of epinephrine doses required for treatment. These results, in addition to previous literature discussed, suggest that the effects of prescribed AH medications may not have a clinically significant impact on the development of epinephrine resistance when treating anaphylaxis in the ED.

## Conclusions

In this study, outpatient use of select AH medications did not show a significant increase in the incidence of refractory anaphylaxis in patients presenting to the ED with an anaphylactic reaction. The effect of outpatient AH medications has not been shown to increase epinephrine requirements for treatment of anaphylaxis. Further exploration regarding the clinical significance of outpatient AH medication use may be warranted. Administration of guideline-recommended medications for the treatment of anaphylaxis should be followed regardless of reported outpatient AH use.
